# Sign epistasis caused by hierarchy within signalling cascades

**DOI:** 10.1038/s41467-018-03644-8

**Published:** 2018-04-13

**Authors:** Philippe Nghe, Manjunatha Kogenaru, Sander J. Tans

**Affiliations:** 1 AMOLF, Science Park 104, 1098 XG Amsterdam, Netherlands; 2 Laboratoire de Biochimie, UMR CBI 8231, ESPCI Paris, PSL Research University, 10 rue Vauquelin, 75005 Paris, France; 3 Delft University of Technology, Bionanoscience department, Van der Maasweg 9, Delft 2629HZ Netherlands; 4 Present Address: Department of Life Sciences, Imperial College London, London, SW7 2AZ United Kingdom

## Abstract

Sign epistasis is a central evolutionary constraint, but its causal factors remain difficult to predict. Here we use the notion of parameterised optima to explain epistasis within a signalling cascade, and test these predictions in *Escherichia coli*. We show that sign epistasis arises from the benefit of tuning phenotypic parameters of cascade genes with respect to each other, rather than from their complex and incompletely known genetic bases. Specifically, sign epistasis requires only that the optimal phenotypic parameters of one gene depend on the phenotypic parameters of another, independent of other details, such as activating or repressing nature, position within the cascade, intra-genic pleiotropy or genotype. Mutational effects change sign more readily in downstream genes, indicating that optimising downstream genes is more constrained. The findings show that sign epistasis results from the inherent upstream-downstream hierarchy between signalling cascade genes, and can be addressed without exhaustive genotypic mapping.

## Abstract (web summary)

Sign epistasis clearly constrains evolution, but its causes are difficult to decipher. Here, the authors study epistasis in a signalling cascade, and arrive at a general criterion and understanding of sign epistasis as arising from the inherent hierarchy between signalling cascade components.

## Introduction

Genetic interactions can render mutational effects, positive or negative on phenotype or fitness, depending upon the genetic background. These sign-changing epistatic interactions are distinct from other forms, such as antagonistic or synergistic epistasis, in which only the magnitude of the mutational effect is changed^1^. Sign epistasis is a central genetic constraint in evolution, as mutations that together are required to evolve a function may be deleterious when they occur individually. This results in a reduced fixation probability, because mutations must occur in particular order or in rapid succession within the same lineage^2^.

Sign epistasis can be detected by systematically combining multiple mutations^3,4^, and has been observed to determine the course of evolution in laboratory experiments^5–7^. Such genotype mapping efforts are laborious and specific to the studied phenotype. It is therefore a central challenge to understand sign epistasis mechanistically. Fisher’s geometric models of phenotype–fitness relations have been used to show that mutations near the fitness peak can display sign epistasis^8–10^, but without addressing molecular mechanisms^11^. Data suggests that sign epistasis can result from direct molecular recognition^2,4,12,13^, and trade-offs between enzymatic activity and thermal stability within single genes^3^. Here we surmised that genes within the signalling cascades could exhibit sign epistasis, as they work in concert to transmit signals faithfully^14^. Single and multiple gene knock-outs have been used to identify such interactions, in order to decipher network connectivity^11^. This approach has allowed to identify redundancies between different pathways^15^ or clustering in metabolic units^16^. However, the role of sign epistasis within the signalling networks and its relation to evolutionary optimisation has been addressed only scarcely^17–20^.

Here, we study pairwise interactions between genes within a signal transduction cascade in *Escherichia coli* using a combinatorial approach. A number of random mutants of *tetR* and *lacI* transcription factors are paired in all combinations to produce a library of cascades, whose ability to transduce signals is quantified by measured input–output relations. These data indicate a significant number of sign epistatic interactions between *tetR* and *lacI*, with the corresponding sign change occurring predominantly for mutations in the downstream *lacI*. To understand these findings, we present an approach to predict epistatic interactions from a biochemical model of the cascade, which relies on the notion that mutations in the repressor LacI do not affect the biochemical parameters of the repressor TetR, and vice versa. This method correctly predicts the observed sign epistasis, the dependence on the amplitude or range of input variations and the regions within phenotype space that display epistasis. Owing to the generality of this geometric analysis, we could show that these results apply to different types of cascades and alternative signal transduction performance measures. Finally, the approach provides a single criterion for the presence of sign epistasis within the full phenotype space: it exists when the optimal value of a phenotypic parameter of one gene depends on a phenotypic parameter that corresponds to another gene, and hence is not affected by the same mutations. This methodology is general and may be applied to any pair of traits that are genetically independent, but contribute in concert to organismal functions or fitness.

## Results

### Measurements of sign epistasis within a signalling cascade

**Fig. 1:**
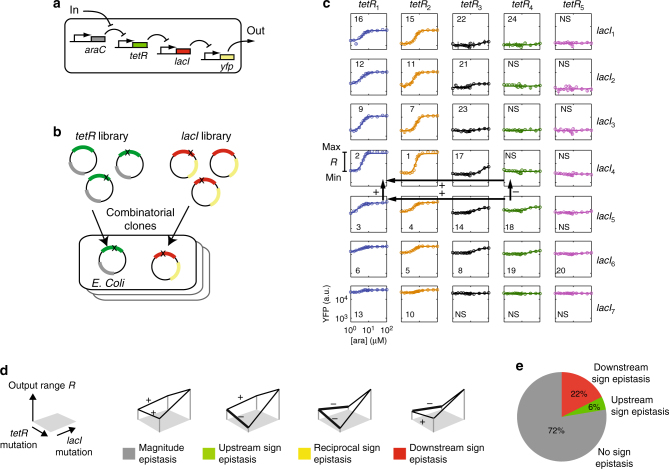
Sign epistasis in combinatorial regulatory cascades. **a** Experimental system: *tetR* and *lacI* genes transduce the arabinose input (in) into a YFP expression output (out). **b** Mutation strategy: randomly mutated *tetR* and *lacI* variants encoded on two separate vector plasmids were transformed in combinatorial fashion into *E*. *coli* cells. **c** YFP versus arabinose responses for all combinations of the five *tetR* and seven *lacI* variants, including the wild-type. Signal transduction ability for each variant is quantified by *R* = out_max_–out_min_ (see *tetR*_1_*–lacI*_4_ variant). Numbers indicate ranking of *R* values for each mutant, with 1 corresponding to the highest *R*, and NS indicates non-significantly increasing responses, compared to measurement noise (*p* < 10^−3^, two-sided Welch *t*-test, Methods section). The four black arrows indicate an example of sign epistasis: *R* decreases when first mutating *lacI*, but increases for all other mutations. Error bars are s.d. over the mean for *n* = 3 biological replicates. *tetR* and *lacI* mutants are ordered by their operator dissociation constant, with the top-left showing the most tightly binding variants. **d** Classes of sign epistasis between *tetR* and *lacI* mutations and associated colour code. Thick lines have a minus sign and indicate decreases in *R*. **e** Fractions of empirically observed epistasis classes for all possible double mutants (pairwise interactions), when *R* is determined over the full arabinose input range. Downstream sign epistasis is predominant

We studied epistasis in a transcriptional signalling cascade in *E*. *coli* (Fig. 1a). This cascade transduces the arabinose concentration signal present in the bacterial environment (input) to a yellow fluorescent protein (YFP) readout (output, Fig. 1a). For two genes within the cascade (*lacI* and *tetR*), we generated mutation variants, using error-prone PCR. We characterised the response of each *lacI* and *tetR* variant individually, using additional fluorescent labels fused in-frame with these genes (Supplementary Fig. 1). Based on these measurements, we selected seven *lacI* variants (*lacI*_1_ to *lacI*_7_) and five *tetR* variants (*tetR*_1_ to *tetR*_5_), including the wild-type forms, that displayed distinct dose-response characteristics (Supplementary Fig. 2–3). Next, we assembled 35 different cascade genotypes, by combining the *tetR* and *lacI* variants (Fig. 1b). This approach allowed us to characterise epistasis between two genes in a transcriptional signalling cascade. Since our aim is to investigate epistasis caused by functional interactions rather than by direct physical contact, we chose regulatory proteins that are well-characterised and known to not interact physically^21,22^.

For each of the resulting genotype, we assessed the signal transduction ability by measuring the YFP response, while varying the arabinose concentration, and quantified the output response range *R* as the difference between the maximum and the minimum YFP output values (Fig. 1c). We consider temporal variations between two environments: one without arabinose, where a minimal output is favoured, and one with arabinose, where a maximal output is favoured. When these environmental variations are fast compared to the typical lifespan of one individual, *R* can be interpreted as a proxy for fitness (Methods section). For a number of cascade genotypes, the output showed no detectable response (ten of the 35 genotypes, *p* < 10^−3^, two-sided Welch *t*-test, Methods section). These genotypes cannot faithfully transduce the signal, as arabinose changes do not produce significant YFP changes. However, these poorly performing cascades can be optimised to achieve higher *R*, through mutations in *tetR* and *lacI*.

The data indicated the combinations of mutations in *tetR* and *lacI*, which exhibit sign epistasis. For instance, the rather weak response of the cascade *tetR*_4_–*lacI*_5_ can be increased by mutating *lacI*_5_ into *lacI*_4_ and *tetR*_4_ into *tetR*_1_ (Fig. 1c, arrows). This optimisation depends strongly on the order in which the mutations occur: *R* decreases further when mutating *lacI* first, then increases when mutating *tetR*. In contrast, *R* increases in both steps, when *tetR* is mutated before *lacI* (Fig. 1c, arrows). The same *lacI* mutation thus can affect *R* positively or negatively, depending on the *tetR* background, which indicates sign epistasis.

More specifically, we refer to this case as downstream sign epistasis (Fig. 1d), because it is the mutation in *lacI* (not in *tetR*) that can have a positive or negative effect on *R*, and *lacI* is downstream of *tetR* within the regulatory cascade. Conversely, we refer to upstream sign epistasis when such a sign change in the mutational effect occurs for the *tetR* mutations. Reciprocal sign epistasis occurs when both *tetR* and *lacI* mutations produce a sign change in *R*. Changes in only the magnitude of *R* were classified as magnitude epistasis (Fig. 1d).

There are 210 independent pairwise interactions to consider in total, given that each of the 35 genotypes can reach 24 distinct genotypes by combining mutations in *lacI* and *tetR*, and all the pairwise interactions comprising the same 4 genotypes are equivalent. We determined all the pairwise interactions and found that a significant fraction displayed some form of sign epistasis. Downstream sign epistasis was the most predominant form (22%), with upstream sign epistasis being a minor fraction (6%), and no case of reciprocal sign epistasis (Fig. 1e).

Functional relations between the repressors may explain the observed epistasis, as their molecular properties must be tuned with respect to each other to transduce signals. However, whether such functional dependencies are indeed sufficient to produce sign epistasis is unclear. For instance, the function of the cascade as a whole depends on a large number of phenotypic parameters, such as binding affinities and cooperativities, while mutations are often pleiotropic and affect more than one parameter at the same time.

### Sign epistasis resulting from geometric fitness models

In order to analyse the origin of the observed sign epistasis, we developed a theoretical approach inspired by geometric fitness models^9,10^. To illustrate the general principles, in this section, we first consider the abstract case of two traits *X* and *Y* whose effects on fitness *F* are expressed by Gaussian functions, which are abstract in the sense that they do not explicitly describe molecular interactions or mechanisms. In the next section, we will apply this approach, using an explicit biochemical function that expresses how *R* depends on LacI and TetR phenotypic parameters, such as repressor dissociation constants.

**Fig. 2:**
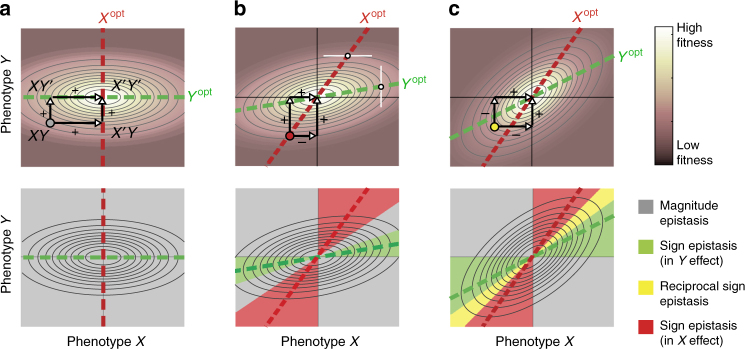
Sign epistasis resulting from geometric fitness models. Diagrams illustrating the principles to determine sign epistasis from phenotype–fitness relations. Here, we consider Gaussian fitness functions that do not specifically describe cascades nor molecular interactions. **a** Non-rotated Gaussian fitness function indicated by colour gradient and elliptical iso-fitness lines. Importantly, we consider mutations that affect *X* but not *Y*, and mutations that affect *Y* but not *X*, which is typically the case if *X* and *Y* have a distinct genetic basis. Mutations in *X* and *Y* are therefore described by orthogonal vectors (arrows). We consider mutations that lead from a suboptimal phenotype *XY* to the optimal *X*′*Y*′(arrows). For any starting phenotype *XY* in this landscape, all four mutations to *X*′*Y'* produce increase in fitness, resulting in magnitude epistasis (grey) everywhere (bottom). **b** Rotated Gaussian fitness function (angle = *π*/12). Starting from the red dot phenotype, one mutation decreases fitness, which implies sign epistasis (see arrows). To understand in mathematical terms, consider the dashed red line (*X*^opt^), which indicates where the tangent of the iso-fitness lines is horizontal (white horizontal line). When moving along this tangent (which changes *X* but not *Y*), the fitness is optimal at *X*^opt^. Thus, starting at or around *X*^opt^, when changing *X* but not *Y*, fitness can only decrease. When the line *X*^opt^ is non-vertical as here, the same mutation can increase fitness in another *Y* background (*Y*’), because the optimum then shifts, and hence the phenotype (*XY’*) no longer lies at the optimum. The same analysis holds for the green dashed line (*Y*^opt^). **c** Rotated Gaussian fitness function (angle = *π*/4). When domains for sign epistasis in *X-* and *Y*-effect (red and green) overlap, one obtains reciprocal sign epistasis (yellow dot). To link these features, such as the degree of rotation or skewness to the molecular level, one can consider a mechanistic fitness function (Fig. 3)

Figure 2a displays a Gaussian fitness function as a function of phenotypes *X* and *Y*. Within this *X*–*Y* plane, mutations can be represented by vectors that point from the original phenotype to the mutant phenotype. An important property of the examined systems is that, the mutations affect either *X* or *Y*, but not both. The corresponding vectors are therefore orthogonal in the *X*–*Y* plane. In our experimental system, mutations in LacI indeed should not affect the phenotypic parameters of TetR, because the two proteins do not interact directly^21,22^. This assumption is further supported by sequence analysis, which did not reveal mutations in the DNA-binding interface of TetR and LacI, and hence no promiscuous operator binding (Supplementary Table 1). Another important feature of this analysis is that, mutants are characterised by phenotypic rather than genetic parameters. Hence, they can be ranked along the corresponding axis (Fig. 2a), also when the mapping between genotype and phenotype is unknown, as is typically the case.

Epistasis is determined by tracking the changes in *F* along the two double-mutation trajectories from *XY* to *X*′*Y*′, in which either *X* or *Y* is mutated first: *XY* → *XY'* → *X*′*Y*′ and *XY* → *X*′*Y*′ → *X*′*Y*′ (Fig. 2, top). Geometrically, these two trajectories together form a rectangle in *X*–*Y* space, with start and end phenotypes located at opposite corners. Here we focus on trajectories ending at the absolute maximum of the landscapes, but the approach can be generalised to other end points (Supplementary Fig. 4b). Considering trajectories starting from all points within the *X*–*Y* plane and ending at the absolute maximum, we colour-coded the initial points, according to the observed epistasis type (Fig. 2, bottom).

In case of a non-rotated Gaussian fitness function (Fig. 2a), one only observes fitness increases along both double-mutation trajectories, for all starting phenotypes. Thus, such phenotype to fitness functions yield exclusively magnitude epistasis (Fig. 2a, bottom). Next, one may consider rotated versions of this landscape (Fig. 2b, c). For moderately rotated landscapes, one observes fitness decreases along the trajectories for some starting phenotypes (Fig. 2b, top), which indicates sign epistasis (Fig. 2b, bottom). Rotating the landscape further leads to reciprocal sign epistasis in addition to sign epistasis (Fig. 2c). Biologically, rotations imply that changing one phenotype can be beneficial in one phenotypic context and deleterious in another^23^, as the optimum value of *X* then depends on *Y*, and vice versa (see below). Similar dependencies were already discussed by Haldane, who noted that “an increase in pigmentation in an animal might be disadvantageous, unless balanced by an increase in the capacity of its liver for storing vitamin D during sunny weather^23,24^.”

To expand on these observations, an optimality perspective is helpful^25^. First, we note that, having a phenotype whose effect on fitness depends on another phenotype does not imply sign epistasis, as seen for the non-rotated Gaussian fitness (Fig. 2a). Instead, the optimum of one phenotype must depend on the value of the other phenotype. We explain this notion in Fig. 2, which shows how one can visually identify the optimum values of *X* by considering the tangent of the iso-fitness lines. At points in the *X*–*Y* space where this tangent is horizontal (Fig. 2b, white line), the value of *X* is optimal for fixed *Y*. These points for different fixed *Y* form a line that we call *X*^opt^ (dashed red lines in Fig. 2). The key is that, phenotypes positioned on the line *X*^opt^ can only decrease the fitness when mutating *X*, as they lie at the optimum. When *X*^opt^ is non-vertical as in Fig. 2b, the same mutation can increase fitness in another *Y* background, because the optimum then shifts and hence the phenotype no longer lies at the optimum. This is seen in Fig. 2b, where the initial point is located close to *X*^opt^ (red dashed line), and hence the *X* mutation decreases fitness, while the same mutation increases fitness after the mutation in *Y*. Conversely, we refer to *Y*^opt^ as the points where the tangent to the iso-fitness lines is vertical (Fig. 2a,c, green dashed lines).

Hence, phenotype-fitness landscapes do not produce sign epistasis when *X*^opt^ and *Y*^opt^ are straight lines that are vertical or horizontal, respectively (Fig. 2a). Conversely, sign epistasis domains do emerge when *X*^opt^ and *Y*^opt^ deviate from such vertical and horizontal lines. There are several uses of such an optimality approach. First, it allows one to define a single criterion to establish the presence of sign epistasis, given a particular phenotype–fitness function. One may alternatively identify sign epistasis by computationally testing pairwise interactions between phenotypes, but this requires exhaustive mapping of high-dimensional spaces and does not allow analytical analysis. In contrast, the parameterised optima approach described here (Fig. 2) allows one to formally describe such a criterion for sign epistasis, and that it applies to phenotypes of arbitrary dimension and a broad category of fitness functions (Supplementary Note 1).

The phenotypic interactions we discuss here should not be confounded with genetic correlations between traits, in which a genetic change affects two traits at the same time. Indeed, the phenotypes *X* and *Y* here are genetically independent—mutations affect *X* or *Y*, but not both. A different situation arises if instead, the latter is not true or unclear for instance, because one considers phenotypes that do not have a distinct genetic basis. Such cases have been addressed previously by theoretical analysis^9,10^. The corresponding mutational vectors are then not necessarily orthogonal to the axis or form a rectangle, as is the case in Fig. 2. Even for skewed landscapes like Fig. 2b, c, this additional freedom then allows trajectories to the optimum that confer fitness increases only, from any point within the phenotype space. Hence one cannot attribute one particular type of epistasis to a point within the phenotype space, which limits the predictive potential. This is specifically the case for associated evolutionary constraint. For instance, the yellow domain of reciprocal sign epistasis implies the existence of phenotypes *XY*, which must first decrease in fitness when moving away from the diagonal, before they can increase again (arrows in Fig. 2c). That is possible, if *X* and *Y* are genetically independent. If not, then this severe evolutionary constraint is not predicted, as it can be broken by moving along the diagonal.

In summary, sign epistasis between genetically independent components can be understood in terms of phenotype–fitness landscape features. In molecular terms, it is not straightforward to identify general rules that lead to rotated landscapes and sign epistasis. However, one can explain and predict epistasis in cases as studied here, where a mechanistic understanding is available, as we will discuss next. We note that for two phenotypes that contribute jointly to fitness, but are *not* independent genetically (unlike the cases considered here), a non-rotated or even a radially symmetric fitness peak may or may not produce sign epistasis^9,10^ as the mutational vectors can then point in any direction. Indeed, the genetic independence of the functionally interacting phenotypes is central to the current prediction of the presence of epistasis, or lack thereof.

### Sign epistasis resulting from a biochemical model

**Fig. 3:**
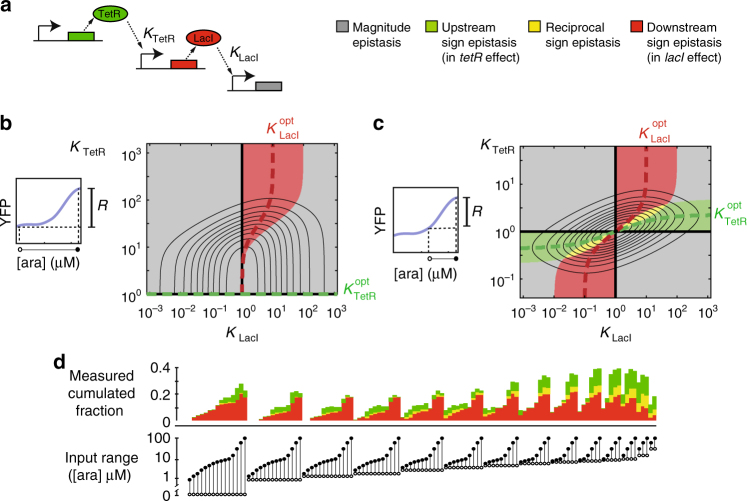
Sign epistasis resulting from a biochemical model and environmental dependence. **a** Simplified diagram of biochemical model of the cascade. This model expresses the output response range *R* as a function of phenotypic parameters and input range (Methods section). Here we indicate *K*_TetR_ and *K*_LacI_, the dissociation constants of each repressor to their operators, but other parameters like Hill coefficients are also considered. Mutations affect either the TetR or the LacI parameters. **b** Theoretical prediction of *R* using the biochemical cascade model (Methods section), for the full 0–100 µM arabinose input range, as a function of *K*_TetR_ and *K*_LacI_. Other parameters like Hill coefficients are fixed at experimentally observed values. Curved grey lines are the corresponding iso-*R* lines. The colour at a particular point indicates the type of epistatic interaction predicted between the phenotype at that point and the optimum phenotype, as in Fig. 2. This optimum is found at the intersection of the axis (black lines). The line $K_{{\mathrm{TetR}}}^{{\mathrm{opt}}}$ indicates the optimal *K*_TetR_ value for fixed *K*_LacI_. $K_{{\mathrm{LacI}}}^{{\mathrm{opt}}}$ indicates the optimal *K*_LacI_ value for fixed *K*_TetR_. $K_{{\mathrm{TetR}}}^{{\mathrm{opt}}}$ is horizontal, resulting in no domain of upstream sign epistasis. $K_{{\mathrm{LacI}}}^{{\mathrm{opt}}}$ is non-vertical, resulting in a domain of downstream sign epistasis (red). **c** Same as **b**, with *R* being computed for a restricted 50–100 µM arabinose input range. Here, the red domain is preserved, while additional green and yellow domains (upstream and reciprocal sign epistasis) are introduced, which is consistent with the empirical data (**d**). **d** Top: stack histogram of empirical epistasis fractions for different input ranges, as determined from the data in Fig. 1c. Each vertical stack corresponding to a different input range. Bottom: corresponding input ranges (open circle: minimum input; full circle: maximum input). The data shows that green and yellow is added to the red fraction, consistent with the predictions (**c**)

In order to apply the above landscape analysis (Fig. 2) for understanding the empirically observed sign epistasis (Fig. 1d), we used a biochemical model of the cascade. The model aims to describe how *R* depends on phenotypic parameters, such as the binding affinity of LacI and TetR for their operators, Hill coefficients, their minimum and maximum expression levels, as well as the range of (arabinose) input values over which *R* is assessed (Methods section). This *R*-function is based on established equations for the equilibrium repression behaviour of transcription factors^26^, and hence serves as a purely theoretical prediction of the functional interactions between LacI and TetR. The idea is to apply the landscape approach (Fig. 2) on this *R*-function to analyse and predict epistatic interactions between LacI and TetR (Fig. 3).

Here we focus on the dependence of *R* on the repressor dissociation constants *K*_TetR_ and *K*_LacI_, while keeping the other phenotypic parameters fixed (Fig. 3a). For the latter, we used values derived by fitting the responses of the separate components, which were measured with additional fluorescent reporters (Supplementary Fig. 5, Supplementary Tables 2 and 3). In the next section, we will consider mutational effects on other phenotypic parameters as well. We found the landscape *R*(*K*_TetR_, *K*_LacI_) to be skewed along one axis: the line $K_{{\mathrm{TetR}}}^{{\mathrm{opt}}}$ does not deviate from the (horizontal) axis, but the line $K_{{\mathrm{LacI}}}^{{\mathrm{opt}}}$ *does* (from the vertical axis). The latter deviation predicts a single epistasis domain (Fig. 3b, red domain), and hence sign epistasis, in which the sign change occurs for mutations in *lacI* and not *tetR*. The latter prediction agrees with the observed predominance of downstream (*lacI*) epistasis in Fig. 1e.

The biochemical model is useful to explain the functional origins of this sign epistasis, as the latter depends on multiple factors and hence is not intuitively understood (Supplementary Fig. 6). The model shows that repression is sensitive only for a window of repressor concentrations near their *K*_d_. For a proper signal transduction, the range of LacI expression levels must overlap with this sensitivity window. A lower *K*_LacI_ (increased affinity) can limit this overlap, as the operator may then always be bound by LacI and hence become insensitive to changes in LacI concentration. However, subsequent decreases in *K*_TetR_ can regain and even increase this overlap, as the increased repression by TetR decreases the LacI concentration and hence operator saturation, while at the same time increasing the range of LacI concentration variations (Supplementary Fig. 6).

The model yielded additional predictions. First, it suggested that the upstream-downstream asymmetry is affected by the range of input values. Specifically, when the input range is restricted, $K_{{\mathrm{TetR}}}^{{\mathrm{opt}}}$ should now also deviate from the (horizontal) axis, leading to an extra domain of upstream sign epistasis, while keeping the downstream sign epistasis domain intact (Fig. 3b, Supplementary Fig. 7 & 8). One can test this assertion by determining the types of epistasis for the pairwise interactions as performed before (Fig. 1e), but now for different ranges of arabinose concentrations (Fig. 3d). The experimental data, indeed showed increased upstream and reciprocal sign epistasis for more restricted arabinose variations, while preserving the downstream sign epistasis (Fig. 3d).

**Fig. 4:**
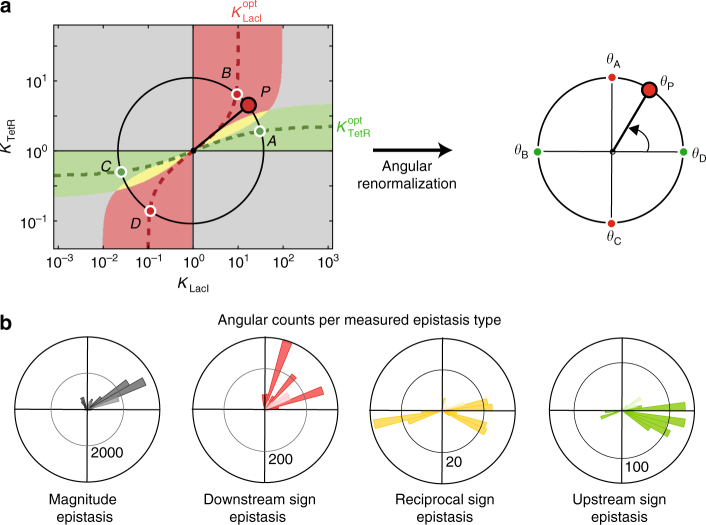
Epistasis domain structure. The biochemical model and epistasis analysis (Fig. 3) suggests a radial epistasis domain structure centred at the optimum, with e.g. downstream sign epistasis (red) in a single domain towards the top and bottom, upstream sign epistasis (green) towards the right and left and reciprocal sign epistasis (yellow) in between (see **a**). Here, we test whether the empirical data are consistent with the domain structure. **a** Angle normalisation scheme, which can be seen as straightening the dashed lines. More specifically, for any point *P*, the angular coordinates $\alpha _{\mathrm{A}}$, $\alpha _{\mathrm{B}}$, $\alpha _{\mathrm{C}}$ and $\alpha _{\mathrm{D}}$ on the black circle passing through *P* are the intersections with the parameterised optima (red and green dashed lines). These angles are normalised to respectively $\theta _{\mathrm{A}} = 0$, $\theta _{\mathrm{B}} = {\mathrm{\pi }}/2$, $\theta _{\mathrm{C}} = {\mathrm{\pi }}$ and $\theta _{\mathrm{D}} = 3{\mathrm{\pi }}/2$. The normalised angle $\theta _{\mathrm{P}}$ for point *P* is interpolated linearly between these angles. **b** Normalised angular histograms of epistasis counts for the empirical data (Fig. 1), aggregated overall input ranges, for all possible initial and final mutants and for all phenotypic parameters. Faded bars: non-significant counts, *p* < 0.05 (one-tailed binomial test, Methods section). Upstream and downstream sign epistasis counts (resp. green and red) are grouped in peaks, which is consistent with the radial organisation of domains (**a**, left). Note that empirical data does not cover the full phenotype space, and hence some angles (e.g. bottom right) are less represented in the data. Some deviations are observed, such as the yellow peak towards the bottom right

Second, the landscape smoothness implied that epistasis types are clustered in phenotypic domains that are arranged in a radial butterfly-like pattern (Figs. 2a, c and 3b, c). To test for this clustering, we mapped the experimental data onto an angular coordinate *θ* (Fig. 4a), as shown previously for Gaussian geometric models^27^. The curved lines, denoting the positions of the restricted optima ($K_{{\mathrm{LacI}}}^{{\mathrm{opt}}}$ and $K_{{\mathrm{TetR}}}^{{\mathrm{opt}}}$), are then mapped onto straight lines with fixed angles (Fig. 4a). This mapping allowed us to consider different input ranges and phenotypic parameters. The resulting angular distributions were indeed peaked (Fig. 4b), as is expected, and hence supports a clustering in radially organised domains. The peaking angles were also consistent, though to larger degree for upstream and magnitude sign epistasis, which point to the right and top-right directions, respectively. The observed angles corresponded to lesser degree to the theoretical expectations for downstream, which not only points to the top, but also to the right, and reciprocal sign epistasis, which points mainly to the right rather than the top right (Fig. 4a, b). Such deviations may have different causes. For instance, the model does not always accurately capture the functional consequences of the mutations, and the studied genotypes do not uniformly sample the full phenotype space.

### Generality of the epistasis domains

Next, we used the biochemical models to examine mutational effects on other phenotypic parameters of the cascade, different cascades that also contain activators, as well as alternative performance criteria.

We first considered the dependence of *R* on the TetR and LacI Hill coefficients, and resulting epistasis domains. This analysis showed a similar radial butterfly pattern, with downstream sign epistasis again along the vertical axis, and upstream sign epistasis along the horizontal axis (Supplementary Fig. 9a). Thus, mutations that affect LacI and TetR Hill coefficients display functional interactions that give rise to sign epistasis. Other pairs of phenotypic parameters also showed a similar epistasis pattern (Supplementary Fig. 9b, c).

**Fig. 5:**
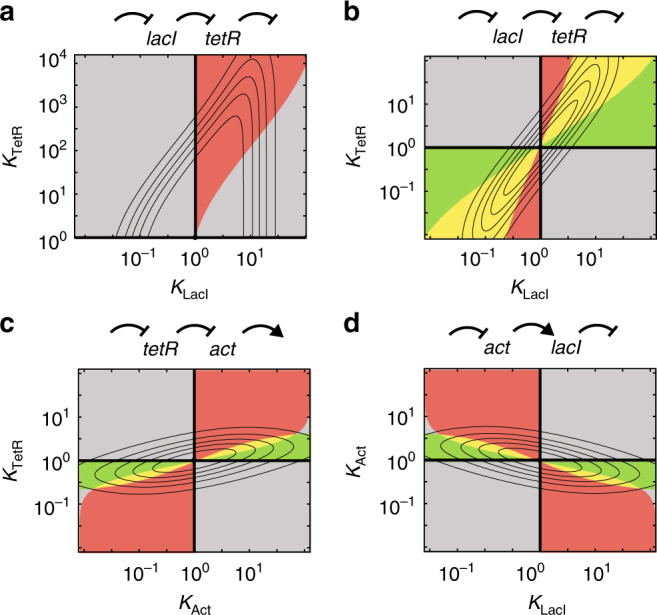
Generality of epistasis domain organisation. Epistasis domains computed for alternative cascade organisations. **a** Epistasis domains for exchanged *tetR*–*lacI* order (*tetR* now downstream), for full input range (0–100 µM arabinose) determined using a biochemical cascade model (Methods section). **b** Same as for **a**, using a restricted input range (50–100 µM arabinose). **a** and **b** show qualitatively the same domain organisation as for the original cascade structure (Fig. 3b, c), indicating that repressor hierarchy is more important than repressor phenotype. **c** Epistasis domains when exchanging *tetR* for a generic activator. **d** Epistasis domains when exchanging *lacI* for a generic activator. **c** and **d** indicate that the case with activators can have a similar domain organisation, though the symmetry can be different (see Supplementary Note 2)

One may also consider cascades in which the hierarchy is inverted, with *lacI* upstream and *tetR* downstream (Fig. 5a, b). For the full range of input values, one then obtains a single domain of downstream sign epistasis, using the biochemical model (Fig. 5a), as in the original cascade (Fig. 3b), though the domain shape differs. Thus, the hierarchy of the cascade components rather than their genetic or phenotypic properties are determining, as the sign change now occurs when mutating *tetR* rather than *lacI*, as *tetR* now is the downstream component. Consistently with the results of the original cascade (Fig. 3c), we find upstream and reciprocal sign epistasis domains added to the phenotype space for more restricted input ranges (Fig. 5b).

Surprisingly, when the downstream *lacI* repressor, in the original cascade, is exchanged with an activator, the organisation of epistasis domains is also similar (Fig. 5c). When instead, the upstream *tetR* is exchanged for an activator, this pattern is simply mirrored against the *Y*-axis (Fig. 5d). These similarities of the epistasis domain organisation for different cascades can be understood from the sensitive window discussed earlier, as it applies to both activators and repressors (see Supplementary Note 2 for formal arguments). Overall, the ubiquitous presence of epistasis in cascades can be understood intuitively from the geometric fitness model analysis, as they emerge whenever the fitness function is skewed and the restricted optima depends on other phenotypic parameters.

We furthermore found that the central results are robust to different fitness measures. We tested fitness functions, based on the input–output fold change, nonlinear cost benefit in variable environments, specific target input–output relations and mutual information quantifying transmission fidelity (Supplementary Fig. 10). The butterfly pattern results whenever the iso-fitness lines are convex (bulge outward away from the origin), which is often the case around fitness peaks in phenotype space. This phenomenon can be seen from the construction of Fig. 2b, where the vertical and horizontal white tangents, which determine the position of the parameterised optima, alternate along the iso-fitness ellipses. Locally concave iso-fitness lines and multipeaked landscapes do display sign epistasis domains, but rather give rise to more complex patterns. Sign epistasis can break down fully if one mutation affects phenotypic parameters of both genes, as performance decrease can then be circumvented. However, pleiotropy within genes does not have such an effect: for instance, sign epistasis domains still emerge in the *K*_TetR_–*K*_LacI_ space, when the changes along the *K*_LacI_ axis simultaneously induces changes in the LacI Hill coefficient (Supplementary Fig. 6d).

## Discussion

At the genotype level, sign epistasis refers to cases in which the sign of a mutational effect depends on the genetic background. Here, we addressed epistasis at the phenotypic level. In similar terms, sign epistasis arises when a change in a phenotype has a positive or negative effect, depending on another phenotype, as long as the two phenotypes are not affected by the same mutations. One can formulate a criterion for this dependence: sign epistasis is observed when one trait affects how another trait can optimally contribute to fitness. This criterion can be used to assess the structure of phenotype spaces as a whole, and hence differs in definitions that consider individual pairs of genotypic or phenotypic change. Moreover, considering epistasis at the phenotypic level in this fashion allows prediction of sign epistasis from functional interactions, and to analyse empirical data that typically do not systematically cover the full phenotype space. These conclusions rely on, and are enabled by, the realisation that phenotypic parameters can be genetically independent. One may expect such genetic independence combined with interactions at the functional level in many cases, for instance, for phenotypic parameters that correspond to distinct genes within networks as considered here.

We used the property of parameter-dependent optima to show that the upstream-downstream hierarchy determines sign epistasis between genes in signalling cascades. This approach could be applied to more complex regulatory networks such as chemotaxis^28^ or osmotic regulation networks^29^, where feedbacks break the notion of hierarchy, but mutations can have independent effects on genetic modules. In these cases, reciprocal regulatory interactions between components could render optima parameter dependent, and thus produce sign epistasis domains within the phenotype space. It remains unclear how the geometric analysis of functional relations could be applied to understand sign epistasis between mutations that have pleiotropic effects on multiple genes or traits. This issue may be addressed by mapping distributions of mutational effects on geometric fitness models, defined by the underlying functional relations between genes or traits.

## Methods

### Construction of the cascade

A synthetic signalling cascade was constructed using *araC*, *tetR* and *lacI* transcription factors along with their cognate promoters. To measure the signal propagation through the intermediate layers and levels of TetR and LacI, we fused these transcription factors with eCFP and mCherry fluorescent reporters, respectively, in a transcriptional-translational fusion with the linker EFLQPGGS. The *tetR-eCFP* and *lacI-mCherry* genes are encoded on two different vector backbones as indicated (Supplementary Fig. 1).

### pAraC-TetR-eCFP

The *tetR* and *eCFP* genes were spliced in a frame, by separating the two genes, by a linker sequence of 24-base pairs (bps), with an overlap PCR. The resulting full-length PCR product was cloned into pAraC vector backbone using EcoRI and XhoI restriction sites, which led to the generation of the pAraC-TetR-eCFP construct. This vector contains a kanamycin-resistant gene for a selection and carries a medium copy *p15A* origin of replication.

### pLacI-mCherry-*P*_*trc*_-eYFP

This construct was created in two step. First, an intermediate p*P*_*trc*_-eYFP vector was constructed as follows: the *P*_*trc*_ promoter, *eYFP* gene and *T0* terminator sequences were spliced by an overlap PCR. The resulting full-length PCR product was cloned utilising XbaI and BbsI restriction enzymes into a vector backbone, containing an ampicillin resistant selection gene and *colE1* origin of replication. In the second step, the *P*_*LtetO1*_ promoter, the *lacI* gene, *mCherry* gene and *T1* terminator were spliced by an overlap PCR. The resulting full-length PCR product was cloned into p*P*_*trc*_-eYFP intermediate vector backbone, using HindIII and XhoI restriction enzymes, to obtain a final pLacI-mCherry-eYFP construct.

### Creation of *tetR* and *lacI* mutants and screening

The mutations were introduced into the coding regions of *tetR* and *lacI* genes by an error-prone PCR, using the Stratagene Genemorph II Random Mutagenesis kit. By following the manufacturer’s protocol, a mutation rate of 1–10 mutations per kb were achieved. The mutagenized PCR amplicons were subjected to restriction digestion, with EcoRI and XmaI or BamHI, and ligated back into the corresponding vector backbones to create mutant libraries. The mutant *tetR* and *lacI* libraries were transformed into *E*. *coli* strain MK01 genetic background by the electroporation procedure. The mutant *tetR* library was co-transformed into MK01 strain, carrying a wild-type encoding *lacI* vector. Similarly, mutant *lacI* library was co-transformed into MK01 strain, carrying a wild-type encoding *tetR* vector. Picked mutant clones were screened in a 96-well plate. The response to external l-arabinose concentration was measured, as explained in the ‘fluorescence measurements’ section, and the resulting measurement data was processed as explained in the ‘data processing’ section. The resulting data is presented in Supplementary Fig. 2 and 3.

### Fluorescence measurements

*E*. *coli* strain MK01^22^ harbouring DNA constructs, were inoculated from a glycerol stock into EZ Rich Defined medium with glucose (Teknova, Hollister, CA, USA, cat. nr. M2105) and appropriate antibiotics. These cultures were grown at 37 °C until early exponential growth phase, and then diluted to a final optical density (OD) of 1 × 10^−4^ at 550 nm (OD550). These diluted cultures were grown in a 96-well optical-bottom black colour microtiter plate (NUNC cat. nr. 165305), containing appropriate antibiotics and inducer concentrations in a total volume of 200 μl per well. The plate was then inserted into Wallac Perkin Elmer Victor^3^ automated plate reader. The OD550 and fluorescence intensities, from three distinct fluorescent proteins (eCFP, mCherry and eYFP), were measured at regular interval of time at 37 °C. This instrument was equipped with a custom-made filter set to record the fluorescence intensities from eCFP (excitation 430/24, emission 480/20), mCherry (excitation 580/20, emission 632/45) and eYFP (excitation 500/20, emission 535/25) fluorescent proteins. To facilitate the bacterial growth, the plate was under double orbital shaking, during the non-reading hours. At every 27 min, 9 μl of sterile water was added to each well to counteract the evaporation.

### Data processing

The raw data of the OD550 and fluorescence intensities were background subtracted from an average value of the wells containing only medium without bacteria. The OD550 values, corresponding to the fluorescence data points, were extrapolated from the logarithmic growth phase data points. The normalised fluorescence was obtained from the exponential growth phase data points, by dividing the derivative of fluorescence by OD550 ([δFluorescence/δt]/[δOD/δt]). The normalised fluorescence for the strain without any construct were averaged and subtracted for the auto-fluorescence from the normalised fluorescence values of stain with constructs. All the analysis steps were performed automatically, using custom-made data processing pipeline in the origin data analysis and graphing software package. Experimental analyses used smooth response ${\mathrm{YFP}}_{{\mathrm{smooth}}} = \frac{{a[{\mathrm{ara}}]^d}}{{b^d + [{\mathrm{ara}}]^d}} + c$ for each mutant (continuous curves of Fig. 1c), fitted on the fluorescence data point with *lsqnonlin* Matlab function for parameters *a, b, c* and *d*.

### Fitness proxy

We considered a variable environment characterised by two input values *x*_1_ and *x*_2_, leading to the respective cascade outputs *y*_1_ and *y*_2_. Each environment has a fitness (or performance) function *F*_1_ and *F*_2_. When these functions follow affine forms of the benefit and cost, $F_1 = a + c{\times}y_1$ and $F_2 = b - c{\times}y_2$. Assuming that an equal time is spent in both environments, the average fitness is $F_{{\mathrm{av}}} = (a + b)/2 - c/2(y_2 - y_1)$. For any (*a*, *b*, *c*), the range $R = y_2 - y_1$ is a direct proxy for relative fitness changes due to mutations, as the latter only affect *y*_1_ and *y*_2_.

### Gaussian fitness function

The centred stretched Gaussian fitness functions rotated of an angle *a* are of the form $F = {\mathrm{exp}}( - (X,Y){{M}}\left( {{{X}},{{Y}}} \right)^{\mathrm{t}})/(2{\mathrm{\pi }}\,{\mathrm{det}}({{M}}))$, where *M* the matrix defined by $m_{11} = \left( {\cos a} \right)^2/2 + 2\left( {\sin a} \right)^2$, $m_{12} = m_{21} = - \sin 2a/4 + \sin 2a$, and $m_{22} = \left( {\sin a} \right)^2/2 + 2\left( {\cos a} \right)^2$.

### Biochemical model and parameters

The kinetic model of the cascade consisted of combining repressors that are described by the following input–output Hill function^26^:

$${\mathrm{TetR}}({\mathrm{ara}}) = \frac{{M_{{\mathrm{AraC}}} - m_{{\mathrm{AraC}}}}}{{1 + \left( {[{\mathrm{ara}}]/{{K}}_{{\mathrm{AraC}}}} \right)^{n_{{\mathrm{AraC}}}}}} + m_{{\mathrm{AraC}}},$$

$${\mathrm{LacI}}({\mathrm{TetR}}) = \frac{{M_{{\mathrm{TetR}}} - m_{{\mathrm{TetR}}}}}{{1 + \left( {{\mathrm{TetR}}/K_{{\mathrm{TetR}}}} \right)^{n_{{\mathrm{TetR}}}}}} + m_{{\mathrm{TetR}}},$$

$${\mathrm{Out}}({\mathrm{LacI}}) = \frac{{M_{{\mathrm{LacI}}} - m_{{\mathrm{LacI}}}}}{{1 + \left( {{\mathrm{LacI}}/K_{{\mathrm{LacI}}}} \right)^{n_{{\mathrm{LacI}}}}}} + m_{{\mathrm{LacI}}},$$

where *M*_i_ is the maximum expression level, *m*_i_ the minimum, *K*_i_ the dissociation constant and *n*_i_ the cooperativity. For activators, we used the functional form

$${\mathrm{Out}} = \frac{{(M_{{\mathrm{Act}}} - m_{{\mathrm{Act}}}) \left( {{\mathrm{in}}/K_{{\mathrm{Act}}}} \right)^{n_{{\mathrm{Act}}}}}}{{1 + \left( {{\mathrm{in}}/K_{{\mathrm{Act}}}} \right)^{n_{{\mathrm{Act}}}}}} + m_{{\mathrm{Act}}}.$$

### Prediction of epistasis

Using the notations above, sign epistasis types were computed using the function *R* = Out(LacI(TetR(ara_min_))) − Out(LacI(TetR(ara_max_))), where ara_min_ and ara_max_ define the bounds of the input range.

### Significance of fitness variations

To assess the typical noise in the response of each mutant, we computed the root mean square deviation $\Delta _{{\mathrm{lac}},{\mathrm{tet}}} = \sqrt {\frac{1}{N}\mathop {\sum }\limits_i ({\mathrm{YFP}}_{\mathrm{i}} - {\mathrm{YFP}}_{{\mathrm{smooth}}})^2}$ between the average ${\mathrm{YFP}}_{\mathrm{i}}$ of triplicates for 16 arabinose values and the smoothed input–output response. To assess the significance of the overall YFP output range *R*_*lac*,*tet*_ of single response curves, we tested the hypothesis '*R*_lac,tet_ is strictly positive' using normally distributed statistics for $\Delta _{{\mathrm{lac}},{\mathrm{tet}}}$ for significance *p* < 0.05. Given that variances between mutants $\Delta _{{\mathrm{lac}},{\mathrm{tet}}}$ are not equal, significance of fitness variations along trajectories was evaluated with a two-sided Welch *t*-test for strict positivity of the fitness change over the full paths (*p* < 0.001, *N* = 44, given the 48 measurement points and four parameters used to fit ${\mathrm{YFP}}_{{\mathrm{smooth}}}$). The same test was used to determine significantly decreasing intermediate steps.

### Significance of angular distributions

To test for the specific angular localisation of each type of epistasis, we considered the null hypothesis that, for all other parameters fixed, epistasis type is attributed randomly, under the constraint that the overall frequency *f*_E_ of each epistasis type is the one measured over the entire sample. Note that the angular renormalisation of mutants is exclusively determined by the fitted values of the experimental binding parameters and the epistasis structure predicted by the model, and are independent from the experimentally measured type of epistasis. Therefore, the null hypothesis is equivalent to drawing epistasis type for each angular bin according to frequencies *f*_E_. We thus tested significance using the binomial distribution $B(N_\theta ,f_{\mathrm{E}})$, where *N*_θ_ is the population of a given angular bin centred on *θ*, with the condition *p* < 0.05 (one-tailed binomial test).

### Code availability

All the codes (Matlab) used in this study are available upon request to the authors.

### Data availability

Data produced for this study is available upon request to the authors.

## Electronic supplementary material

Supplementary Information(PDF 2150 kb)
