# Supplementary material for: Sign epistasis caused by hierarchy within signalling cascades
Source: Nat Commun. 2018 Apr 13;9:1451. doi: 10.1038/s41467-018-03644-8 (PMC5899173; doi:10.1038/s41467-018-03644-8)
Supplement: Supplementary file 1 — Supplementary Information(PDF 2150 kb) [file 41467_2018_3644_MOESM1_ESM.pdf]

Supplementary Information

Sign epistasis caused by hierarchy within signalling cascades

Nghe et al.

Supplementary Note 1: Analysis of sign epistasis based on parameterized optimization

Notations and definitions

Phenotypic parameters: $\mathbf{X}$ and $\mathbf{Y}$ are vectors corresponding to two respective sets of phenotypic parameters of arbitrary dimension. Note that in the main text we only consider the case of scalars denoted X and Y. We consider mutations that affect one or several parameters within a given set, but that do not affect $\mathbf{X}$ and $\mathbf{Y}$ parameters at the same time. Experimentally, indexed vectors $\mathbf{X_i}$ and $\mathbf{Y_j}$ correspond to the properties of the mutant (i,j) of repressors *lacI*$_\mathrm{i}$ and *tetR*$_\mathrm{j}$ (Supplementary Tables 2 and 3): $\mathbf{X_i} = (K_\mathrm{i}^\mathrm{lac}, h_\mathrm{i}^\mathrm{lac}, m_\mathrm{i}^\mathrm{lac})$ and $\mathbf{Y_j} = (K_\mathrm{j}^\mathrm{tet}, h_\mathrm{i}^\mathrm{tet}, m_\mathrm{j}^\mathrm{tet})$, where K_i^a is the dissociation constant of protein a, h_i^a the cooperativity and m_i^a the minimum expression level (also referred to as leakage expression) of gene a.

Parameterized optima: Given a fitness function F that depends on $\mathbf{X}$ and $\mathbf{Y}$, we define the parameterized optima $\mathbf{X}^\mathrm{opt}(\mathbf{Y})$ as the value of $\mathbf{X}$ that gives the maximum F for fixed Y [1]. Similarly, $\mathbf{Y}^\mathrm{opt}(\mathbf{X})$ is the optimal value of $\mathbf{Y}$ given a fixed $\mathbf{X}$. $\mathbf{X}^\mathrm{opt}$ and $\mathbf{Y}^\mathrm{opt}$ are the loci, in the parameter space, of the maxima of F restricted to the other parameter set. When $\mathbf{X}$ and $\mathbf{Y}$ consist of single parameters, $\mathbf{X}^\mathrm{opt}$ and $\mathbf{Y}^\mathrm{opt}$ are lines.

Variations in the parameterized optima: This refers to cases where the parameterized optima $\mathbf{X}^\mathrm{opt}(\mathbf{Y})$ or $\mathbf{X}^\mathrm{opt}(\mathbf{X})$ are not constant. As the manifold $\mathbf{X}^\mathrm{opt}$ necessarily goes through the point $(\mathbf{X}_\mathrm{max}, \mathbf{Y}_\mathrm{max})$, variations in the parameterized optima can be observed as deviations of this manifold from the hyperplane defined by $\mathbf{Y}$ constant and going through $(\mathbf{X}_\mathrm{max}, \mathbf{Y}_\mathrm{max})$. If fitness is derivable, another condition is that any of the partial derivatives $\partial x_k^{opt}/\partial y_l$ is not uniformly equal to zero.

Epistasis along trajectories leading to the fitness optimum

We aim to determine a general criterion for observing sign-epistasis within the phenotype space of the coordinates of $\mathbf{X}$ and $\mathbf{Y}$. Consider double mutation trajectories beginning at an arbitrary point $(\mathbf{X_1}, \mathbf{Y_1})$ and ending at the fitness maximum $(\mathbf{X}_\mathrm{max}, \mathbf{Y}_\mathrm{max})$. We consider mutations that are not phenotypically neutral: $\mathbf{X_1} \neq \mathbf{X}_\mathrm{max}$ and $\mathbf{Y_1} \neq \mathbf{Y}_\mathrm{max}$. The two possible trajectories are: $(\mathbf{X_1}, \mathbf{Y_1}) - (\mathbf{X}_\mathrm{max}, \mathbf{Y_1}) - (\mathbf{X}_\mathrm{max}, \mathbf{Y}_\mathrm{max})$ and $(\mathbf{X_1}, \mathbf{Y_1}) - (\mathbf{X_1}, \mathbf{Y}_\mathrm{max}) - (\mathbf{X}_\mathrm{max}, \mathbf{Y}_\mathrm{max})$. Let us focus on the first trajectory, when mutating $\mathbf{X}$ then $\mathbf{Y}$. There exist initial points $(\mathbf{X_1}, \mathbf{Y_1})$ such that $F(\mathbf{X_1}, \mathbf{Y_1}) > F(\mathbf{X}_\mathrm{max}, \mathbf{Y_1})$ if and only if $\mathbf{X}^\mathrm{opt}(\mathbf{Y_1}) \neq \mathbf{X}_\mathrm{max}$. The backward direction of this implication is due to $F(\mathbf{X}^\mathrm{opt}(\mathbf{Y_1}), \mathbf{Y_1}) > F(\mathbf{X}_\mathrm{max}, \mathbf{Y_1})$, by definition of $\mathbf{X}^\mathrm{opt}$ as the maximum of $F(\mathbf{X}, \mathbf{Y_1})$ for fixed $\mathbf{Y_1}$. The forward implication is due to $\mathbf{X}^\mathrm{opt}(\mathbf{Y_1}) = \mathbf{X}_\mathrm{max}$ implying $F(\mathbf{X_1}, \mathbf{Y_1}) \leq F(\mathbf{X}_\mathrm{max}, \mathbf{Y_1})$ for all $\mathbf{X_1} \neq \mathbf{X}_\mathrm{max}$. Now, if we do not fix $\mathbf{Y_1}$ and ask whether there exist arbitrary initial points which display a type of sign epistasis, the reasoning above shows that it is necessary and sufficient to find a single $\mathbf{Y_1}$ such that $\mathbf{X}^\mathrm{opt}(\mathbf{Y_1}) \neq \mathbf{X}_\mathrm{max}$. As this overall applies when exchanging the role of $\mathbf{X}$ and $\mathbf{Y}$, we have:

> Consider mutations affecting independently two vectors of phenotypic parameters $\mathbf{X}$ and $\mathbf{Y}$. There exists sign epistasis between mutations in $\mathbf{X}$ and $\mathbf{Y}$ that lead to the fitness maximum if and only if the optimal value of $\mathbf{X}$ varies with $\mathbf{Y}$, or vice versa.

Epistasis domains

Here we explore how phenotypes displaying epistasis are organized within phenotype space. Suppose now that $\mathbf{X}^\mathrm{opt}$ indeed varies. If F is continuous, this implies the existence

of a neighbourhood $\delta_{\mathbf{X}}$ which has the dimension of the full space, such that for all $(\mathbf{X_1}, \mathbf{Y_1})$ in $\delta_{\mathbf{X}}$, $F(\mathbf{X_1}, \mathbf{Y_1}) > F(\mathbf{X}_{\max}, \mathbf{Y_1})$. We call $D_{\mathbf{X}}$ the maximal connected domain over which this inequality is verified, which we have just shown to be non-empty, and which contains $\mathbf{X}^{\mathrm{opt}}(\mathbf{Y}) \neq \mathbf{X}_{\max}$ for all $\mathbf{Y}$. Note furthermore $\mathbf{X}^{\mathrm{opt}}$ splits the domain $\mathrm{D}_{\mathbf{X}}$ in two parts. We similarly define D_{Y}, which is non-empty if and only if $\mathbf{Y}^{\mathrm{opt}} \neq \mathbf{Y}^{\max}$. These domains allow to classify epistasis patterns for trajectories leading to the fitness maximum:

- If $\mathrm{D}_{\mathbf{X}}$ and $\mathrm{D}_{\mathbf{Y}}$ overlap, double mutant trajectories that begin in $\mathrm{D}_{\mathbf{XY}} = \mathrm{D}_{\mathbf{X}} \cap \mathrm{D}_{\mathbf{Y}}$ and end at the fitness maximum display reciprocal sign epistasis.
- For initial point in $\mathrm{D}_{\mathbf{X}} \backslash \mathrm{D}_{\mathbf{Y}}$ (resp. $\mathrm{D}_{\mathbf{Y}} \backslash \mathrm{D}_{\mathbf{X}}$, where the backslash symbol is the set difference), double mutant trajectories ending at the maximum display sign epistasis due to a fitness decrease when first mutation **X** (resp. **Y**).

We illustrate the above construction in Supplementary Figure 4a, for the case of the rotated Gaussian of main text Fig 2b. In this example, **X** and **Y** each consist of a single parameter, then denoted X and Y, which for example could respectively be the dissociation constants of our repressors K_{lacI} and K_{tetR}. Let us fix the value of Y as represented by the white dotted line L_Y. Along this line, we note three particular X coordinates: $X_{\mathrm{end}} = X_{\max}$ which is invariant and is the X coordinate of the end point, $X^{opt}(Y)$ which is the point where fitness is maximum along L_Y, and X_{edge} which is such that $F(X_{\mathrm{edge}}, Y) = F(X_{\max}, Y)$. Given the convexity of the iso-fitness lines, all trajectories beginning on the segment $[X_{\max}, X_{\mathrm{edge}}]$ for a given Y will have sign epistasis to the maximum. When X^{opt} is a straight line overlapping the Y-axis and going through $X_{\max}$ (as for the non-tilted Gaussian, main text Fig. 2a), we have $X_{\max} = X^{\mathrm{opt}}(Y) = X_{\mathrm{edge}}$. Then D_X is empty and there is no sign epistasis due to mutations in X. As the same applies for Y, there is no sign epistasis in Y for a non-tilted Gaussian. For the Gaussian tilted by $\pi/4$ (main text Fig. 2c), D_X and D_Y overlap, so that D_{XY} is non empty leading to reciprocal sign epistasis.

Trajectories ending at an arbitrary point

Here we consider trajectories for which the end phenotype $(\mathbf{X}_{\mathrm{end}}, \mathbf{Y}_{\mathrm{end}})$ is not the fitness maximum $(\mathbf{X}_{\max}, \mathbf{Y}_{\max})$. One consequence is that there are some starting phenotypes for which the fitness is higher than the end phenotype (domain S_0), and hence neither **X** or **Y** are optimized. For consistency, these trajectories are excluded, and we refer to these as "forbidden" domains. Similarly, only **X** or **Y** may not be optimized (forbidden domains $S_{\mathbf{X}}$ or $S_{\mathbf{Y}}$, Supplementary Fig. 4b). Outside the forbidden domains, the existence of variable parameterized optima is a sufficient condition for the existence of sign epistasis. Note that it is not a necessary condition here anymore, as it now possible to construct landscapes with specific asymmetries causing sign epistasis for trajectories that cross the fitness optimum in both directions. However the domain analysis defined by the parameterized optima still applies (ex: X_{edge} defined by $F(X_{edge}, Y) = F(X_{end}, Y)$ in Supplementary Fig. 4b).

Higher dimensional analysis

The above analysis of the relation between parameterized optima and sign epistasis is valid for parameter vectors $\mathbf{X} = (x_1, \ldots, x_{\mathrm{I}})$ and $\mathbf{Y} = (y_1, \ldots, y_{\mathrm{J}})$ of any dimensionalities I and J. In particular, the existence of variations in a single parameter x_{i} with another single parameter y_{j} is a sufficient condition for the existence of sign epistasis. It is possible that several pairs $(x_{\mathrm{i}}, y_{\mathrm{j}})$ display variations in their parameterized optima for all other parameters fixed to the value of the end point of the trajectory. We verified this was indeed the case using relevant pairs of fitted parameters (Supplementary Tables 2 and 3,

Supplementary Fig. 5) of the cascade model $(K^{\text{tet}}, K^{\text{lac}})$, $(n^{\text{tet}}, K^{\text{lac}})$, $(K^{\text{tet}}, n^{\text{lac}})$, $(n^{\text{tet}}, n^{\text{lac}})$, as shown in Supplementary Fig. 9.

We now introduce a method to examine the spatial organization of epistasis domains in sub-spaces, such as 2D planes within a 5D parameter space. We discuss at the end of this section the implications of this method for our angular renormalization scheme used in main text Figure 4. The analysis of sub-spaces is important for studies where not all phenotypic parameters can be characterized and hence are hidden, which may very well be the case in general.

We note $(\mathbf{X}, \mathbf{Y}) = (\mathbf{x}, \bar{\mathbf{x}}, \mathbf{y}, \bar{\mathbf{y}})$, where $(\mathbf{x}, \mathbf{y})$ are the coordinates in the lower dimensionality space P, and $(\bar{\mathbf{x}}, \bar{\mathbf{y}})$ are the hidden coordinates. Consider a double swap from $(\mathbf{X}, \mathbf{Y})$ to $(\mathbf{X}_{\text{end}}, \mathbf{Y}_{\text{end}})$, where $(\bar{\mathbf{x}}, \bar{\mathbf{y}})$ can change and hence the end point can be outside P. We note $F_{|\text{P}}$ the restriction of the fitness function F to P. We define the "restricted parameterized optima" $\mathbf{x}_{|\text{P}}^{\text{opt}}(\mathbf{y})$ as the $\mathbf{x}$-values where $F_{|\text{P}}(\mathbf{x}, \mathbf{y})$ is maximal for fixed $\mathbf{y}$, while $(\bar{\mathbf{x}}, \bar{\mathbf{y}})$ is also fixed. A similar definition applies for $\mathbf{y}_{|\text{P}}^{\text{opt}}(\mathbf{x})$. What we show here is that epistasis domains in P are located around the restricted parameterized optima (in P), even though, as mentioned, the mutations can confer changes in hidden parameters $(\bar{\mathbf{x}}, \bar{\mathbf{y}})$. Specifically, if there exist sign epistasis in P, there must exist an initial point $(\mathbf{x}, \bar{\mathbf{x}}, \mathbf{y}, \bar{\mathbf{y}})$ such that $F(\mathbf{x}, \bar{\mathbf{x}}, \mathbf{y}, \bar{\mathbf{y}}) > F(\mathbf{x}_{\text{end}}, \bar{\mathbf{x}}_{\text{end}}, \mathbf{y}, \bar{\mathbf{y}})$. As by definition $F_{|P}(\mathbf{x}_{|\text{P}}^{\text{opt}}, \mathbf{y}) > F_{|\text{P}}(\mathbf{x}, \mathbf{y})$, we have a fortiori $F(\mathbf{x}_{|\text{P}}^{\text{opt}}, \bar{\mathbf{x}}, \mathbf{y}, \bar{\mathbf{y}}) > F(\mathbf{x}_{\text{end}}, \bar{\mathbf{x}}_{\text{end}}, \mathbf{y}, \bar{\mathbf{y}})$. In other words:

The restriction of an epistasis domain to a sub-space P necessarily intersects one of the restricted parameterized optima computed in P.

We have that: (i) restricted parameterized optima $\mathbf{x}_{|\text{P}}^{\text{opt}}$ and $\mathbf{y}_{|\text{P}}^{\text{opt}}$ are determined by the fitness function $F_{|\text{P}}$, but not by the end point of the double mutant trajectories, and (ii) epistasis domains are constrained to be localized around these restricted parameterized optima. Consequently, a radial alternation of $\mathbf{x}_{|\text{P}}^{\text{opt}}$ and $\mathbf{y}_{|\text{P}}^{\text{opt}}$ imposes a corresponding radial ordering of the sign epistasis domains, which is the same whatever the end point considered. In the case of our regulatory cascade, the radial alternation of restricted parameterized optima in $(K^{\text{tet}}, K^{\text{lac}})$ planes imposes the stereotypical "butterfly" sign epistasis patterns for starting points of double mutant trajectories, whatever the values of the other parameters (cooperativity, leakage) and even when they vary.

Supplementary Note 2: Mathematical analysis of activator-repressor symmetries

We demonstrate mathematically the symmetries and invariances observed when exchanging the role of repressors and activators for standard thermodynamics-based models of regulatory modules [2]. In these models: (1) if C is the concentration of the regulatory protein and X the dissociation constant to its binding site, the regulatory response is a function of C/X [2]; (2) There exist an increasing function H such that activator and repressor responses are of the form $H_x^+(c) = H(c - x)$ or $H_x^-(c) = H(-(c - x))$, where c and x the logarithms of C and X. From this point, all values will be referred to in the logarithmic scale. Let $I = [a, b]$ be the input interval of the cascade and call J the concentration range of the upstream gene in the cascade. Given that H is monotone, the output of the first regulatory component verifies:

$$\begin{aligned} J = H_x^+(I) &= [H_x^+(a), H_x^+(b)] = [H(a - x), H(b - x)] \\ &= [H(-(b + x) + a + b), H(-(a + x) + a + b)\,] \\ &= [H_{-x+a+b}^-(b), H_{-x+a+b}^-(a)] = \; H_{-x+a+b}^-(I). \end{aligned}$$

The output interval J of the first element is thus the same whether the first component is H_x^+ or H_{-x+a+b}^-. As this invariance is independent of the downstream component using J as an input, this shows the mirror symmetry $(x - \bar{x} \rightarrow -x - \bar{x})$ of axis y at $\bar{x} = -(a + b)/2$, applied to the first binding parameter when changing the nature of the first element of the cascade.

Now, we study mutations that change the dissociation constant y of the downstream component of the cascade. We can adapt the reasoning above to the downstream gene and show that for each given x, there exists a translation $\bar{y}_x$ such that $R^+(x, y - \bar{y}_x\,) = R^-(x, -y - \bar{y}_x)$, which respectively describe the cascade output range when the second component is an activator or a repressor. However, unlike in the paragraph above, we have to take into account the dependence of the interval J on the variations of the parameter x of the first component and note J_x. Due to the monotonicity of the regulatory response and its saturations for arbitrary low and high input, the amplitude of the output of the first component $\max(J_x) - \min(J_x)$ is a function that increases from 0 for arbitrary small x, to a maximum for a certain x^*, and decreases back to 0 for arbitrary large x. Consequently, by the intermediate value theorem for continuous functions, there exists a function s that associates to every $x < x^*$ a unique $s(x) > x^*$ such that $\max(J_x) - \min(J_x) = \max(J_{s(x)}) - \min(J_{s(x)})$. The output range of the cascade R then verifies $R(x, y - m(x)) = R(s(x), y - m(s(x)))$ for all y, where *m(x)* is the middle of the interval J_x. Bringing these properties together, we obtain for all (x, y):

$$R^+\big(x, y - \bar{y}_x - m(x)\big) = R^-\big(x, -y - \bar{y}_x - m(x)\big) = R^+(s(x), y - \bar{y}_{s(x)} - m(s(x))) = R^-(s(x), -y - \bar{y}_{s(x)} - m(s(x))).$$

We have shown that thermodynamic models of repressors and activators display 3 symmetries which lead to the apparent invariance when changing the nature of the second component: (S1) *x* and *s(x)* are symmetric relative to x^* on the *x* axis, (S2) *m(x)* and *m(s(x))* are symmetric relative to *m(x*)* on the y axis, and (S3) $R(x, y)$ is invariant relative to *m(x)* for fixed *x*. Symmetries S1 and S2 can be verified in the general case given elementary transformations applied to the phenotype-fitness landscape. Indeed, any arbitrary and monotonic transformation that independently applies to the x or y axis results in stretching the epistasis in one direction of the other, without affecting its nature. Therefore, such transformations allow to recover S1 and S2 without affecting the relative position of epistasis domains. Although the invariance S3 is specific to the shape of the response used in this model, S3 is a point symmetry in the general case. Indeed, the effect of changing the second component from activator to repressor leads to a rotation of π of centre (x*, m(x*)).

To generalize the analysis to two arbitrary components taken within a cascade of arbitrary length, one can simply replace the function that describes one of the cascade component with a function describing a larger cascade. For instance the composition between two successive repressors is overall equivalent to an activator [3]. The latter function will be monotonous if –as we assume here- the components of that larger cascade are monotonous. The analysis will thus remain similar, as it requires only monotonicity, and does not depend on the precise functional form.

Mutant	Gene	Protein
tetR#15	G185T	R62M
	C248T	A83V
	A347T	Q116L
tetR#31	G160C	A54P
	C363T	Silent
	T543A	Silent
tetR#34	C196G	H66D
	A572T	E191V
tetR#46	A135G	Silent
	T234C	Silent
	G501A	Silent
	G566C	G189A
lacI#03	G406A	V136M
	C420T	Silent
	G590A	R197H
	G852A	Silent
	T920C	L307P
	G1057C	V353L
lacI#06	G31A	E11K
	A626G	Q209R
	T668C	M223T
	A802G	I268V
lacI#20	T143C	I48T
	C907A	R303S
lacI#23	A55T	T19S
	C85A	H29N
	T344A	L115H
	G427A	V143I
	A466T	I156F
	T506A	L169Q
	C682G	Q228E
	T887A	L296Q
	T1014A	Silent
lacI#31	T529A	L177M
	A536C	H179P
	T565C	Silent
	Δ-17A	
lacI#35	A116T	E39V
	A161G	Q54R
	A304G	S102G
	C417A	Silent

Supplementary Table 1: **Genotypes of *tetR* and *lacI* mutants.** The mutant encoding vectors were isolated using agarose gel electrophoresis, and subjected to Sanger sequencing after amplification and re-transforming into the MK01 strain.

	K_{tetR}/M_{araC}	e_{90}	n_{tetR}	e_{90}
$tetR_1$	$2{,}2.10^{-2}$	$\pm 0{,}26.10^{-2}$	2.3	±0,2
$tetR_2$	$5{,}7.10^{-2}$	$\pm 0{,}55.10^{-2}$	3.5	±0,5
$tetR_3$	$2{,}0.10^{-1}$	$\pm 0{,}20.10^{-1}$	1.8	±0,2
$tetR_4$	$5{,}0.10^{-1}$	$\pm 0{,}3.10^{-1}$	2.3	±0,5
$tetR_5$	1,2	±0,7	2.1	±1,7

Supplementary Table 2: *tetR* fitting parameters. The column e_{90} refers to 90% confidence intervals.

	K_{lacI}/M_{tetR}	e_{90}	n_{lacI}	e_{90}	m_{lacI}/ M_{lacI}	e_{90}
$lacI_1$	$3{,}4.10^{-4}$	$\pm 0{,}71.10^{-4}$	0,42	±0,02	0	-
$lacI_2$	$1{,}8.10^{-3}$	$\pm 0{,}31.10^{-3}$	0,46	±0,03	0	-
$lacI_3$	$3{,}2.10^{-3}$	$\pm 0{,}43.10^{-3}$	0,64	±0,07	$1{,}0.10^{3}$	$\pm 0{,}4.10^{3}$
$lacI_4$	$5{,}5.10^{-2}$	$\pm 0{,}51.10^{-2}$	2,0	±0,33	$2{,}8.10^{3}$	$\pm 0{,}6.10^{3}$
$lacI_5$	$4{,}6.10^{-2}$	$\pm 0{,}55.10^{-2}$	0,43	±0,02	0	-
$lacI_6$	$1{,}6.10^{-1}$	$\pm 0{,}11.10^{-1}$	0,42	±0,02	0	-
$lacI_7$	2,7	±0,50	0,31	±0,03	0	-

Supplementary Table 3: *lacI* fitting parameters. We fitted all mutants with the 3 free parameters including the minimum expression level. When this level was not significantly different from 0, we refitted the corresponding mutant with a 2 parameters model, imposing $m_{lacI}=0$.

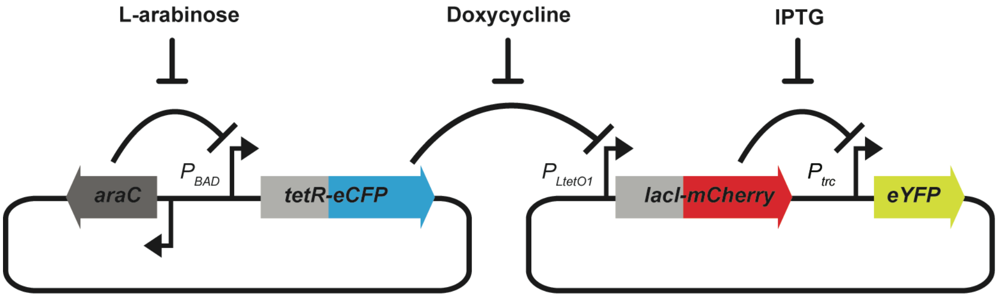

Supplementary Figure 1: Schematic diagram of the studied signalling cascade. The cascade constitutively expresses *araC* from a bi-direction promoter P_{BAD}. In absence of L-arabinose inducer, the resulting AraC transcription factor represses transcription of downstream *tetR, which* is tagged with the *eCFP* fluorescent marker. In absence of Doxycycline, TetR represses transcription from the P_{Ltet01} promoter. The downstream LacI, which is tagged to the mCherry fluorescent marker, represses the transcription of *eYFP* from P_{trc} promoter in absence of isopropyl-β-D-galactopyranoside (IPTG).

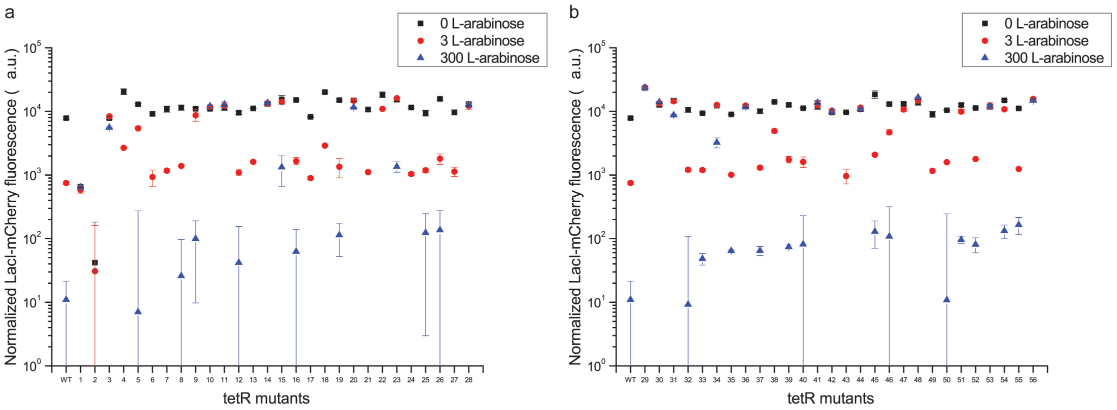

Supplementary Figure 2: Phenotypes of *tetR* mutants. Expression of downstream *lacI-mCherry* gene was measured for three L-arabinose conditions (in μM) as indicated. A wild-type encoding *tetR* vector was also measured along with the mutant *tetRs* and is displayed as a 'WT' as reference. The error bars represent the standard deviation over the mean across the 2 biological replicates. (a) Display mutant clones from 1 to 28, while (b) display 29 to 56. Mutants #15, #31, #34 and #46 were used in this study.

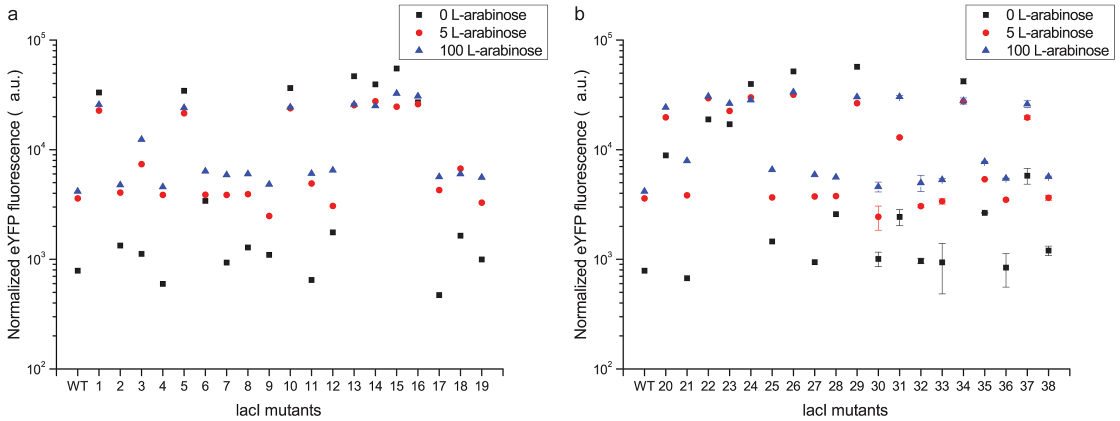

Supplementary Figure 3: Phenotypes of mutant *lacI* clones. Expression of the downstream *eYFP* gene was measured in three L-arabinose conditions (in µM) as indicated. A wild-type encoding *lacI* vector was also measured along with the mutant *lacIs* and is displayed as a 'WT' for a reference. The error bars represent the standard deviation over the mean across the 2 biological replicates. (a) Display mutant clones from 1 to 19, while (b) display 20 to 38. Mutants #03, #06, #20, #23, #31 and #35 were chosen for further study.

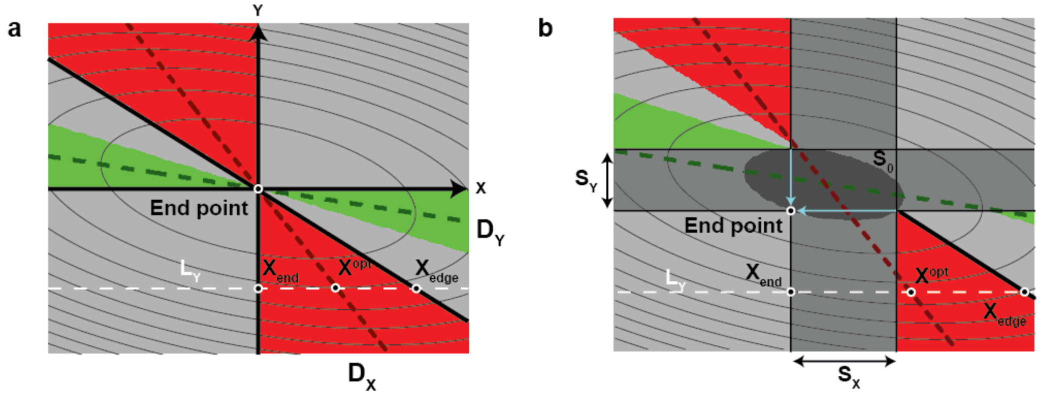

Supplementary Figure 4: Graphical analysis of sign-epistasis in a tilted Gaussian phenotype-to-fitness model. *X* and *Y* coordinates are phenotypic parameters of two distinct genes, such as a dissociation constants. The grey ellipses are iso-fitness lines of a Gaussian fitness function that depends on *X* and *Y,* and is tilted by π/12. The red domains D_X consists of all the starting phenotypes for which the fitness decreases when mutating *X* first, *Y* second, and then ending at the fitness maximum (**panel a**), or another arbitrary phenotype (**panel b**). The green domain D_Y consists of starting phenotypes that yield a fitness decrease when mutating *Y* first. Parameterized optima X^{opt} and Y^{opt} are represented as darker dashed lines. When the end point is not the optimum (panel b), some starting phenotype domains S_x and S_y are the respectively vertical and horizontal grey stripes, S_0 being the darker grey ellipse.

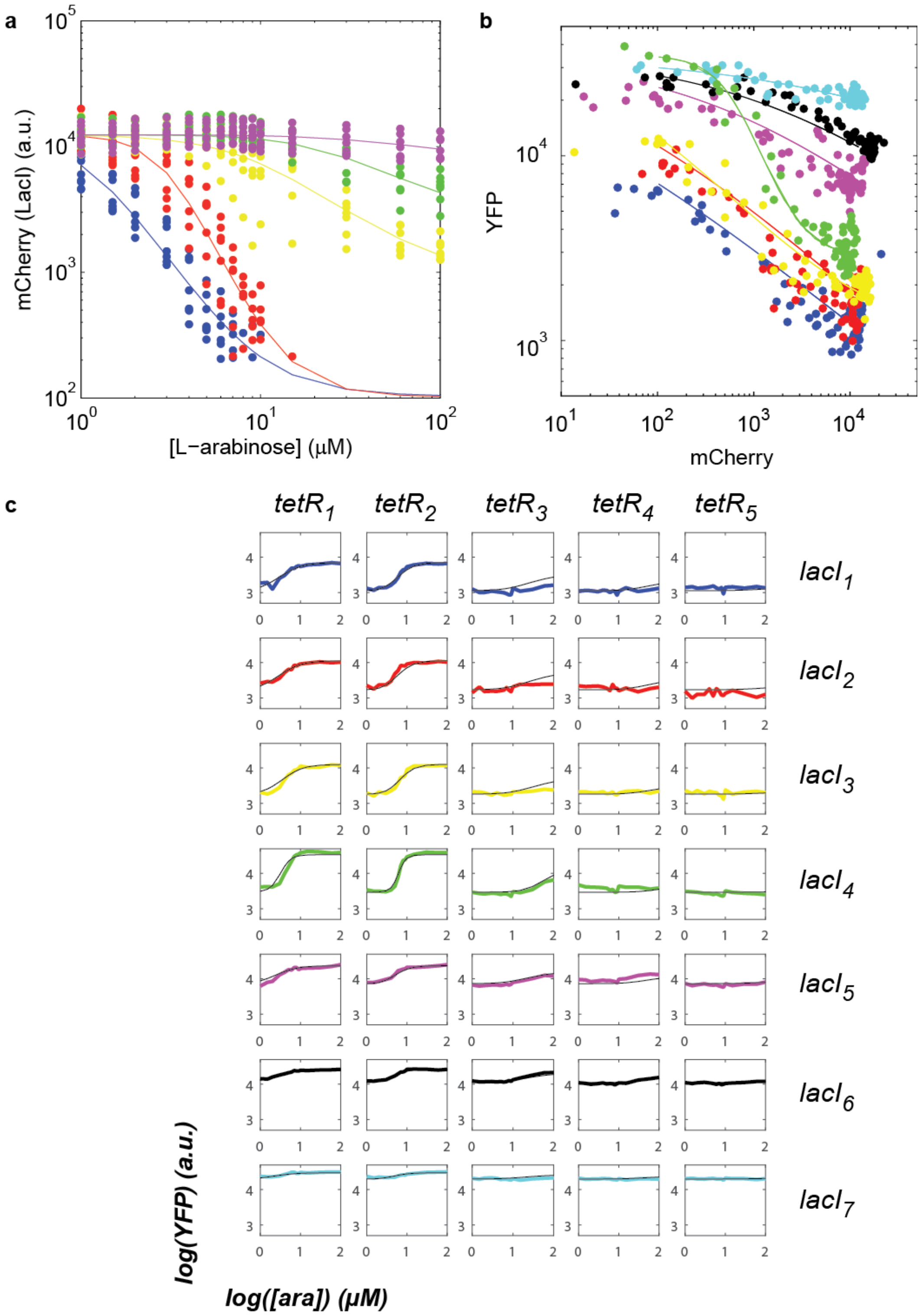

Supplementary Figure 5: fitting of the response curves of the cascade. a) Fits of *tetR* mutants. Note that different lacI mutants have the same color; thus the observed variability for a given arabinose input reflect these genetic differences rather than measurement error. The limited variability shows that the *lacI* genetic changes have only limited effect on the response of the *tetR* mutants. b) Fits of *lacI* mutants. Similarly, different *tetR* mutants have the same colour. c) Experimental responses of the full cascade (coloured lines) and theoretical responses from the mathematical model (black lines, Methods) using the separately fitted *tetR* and *lacI* parameters (panels a and b), without additional fitting.

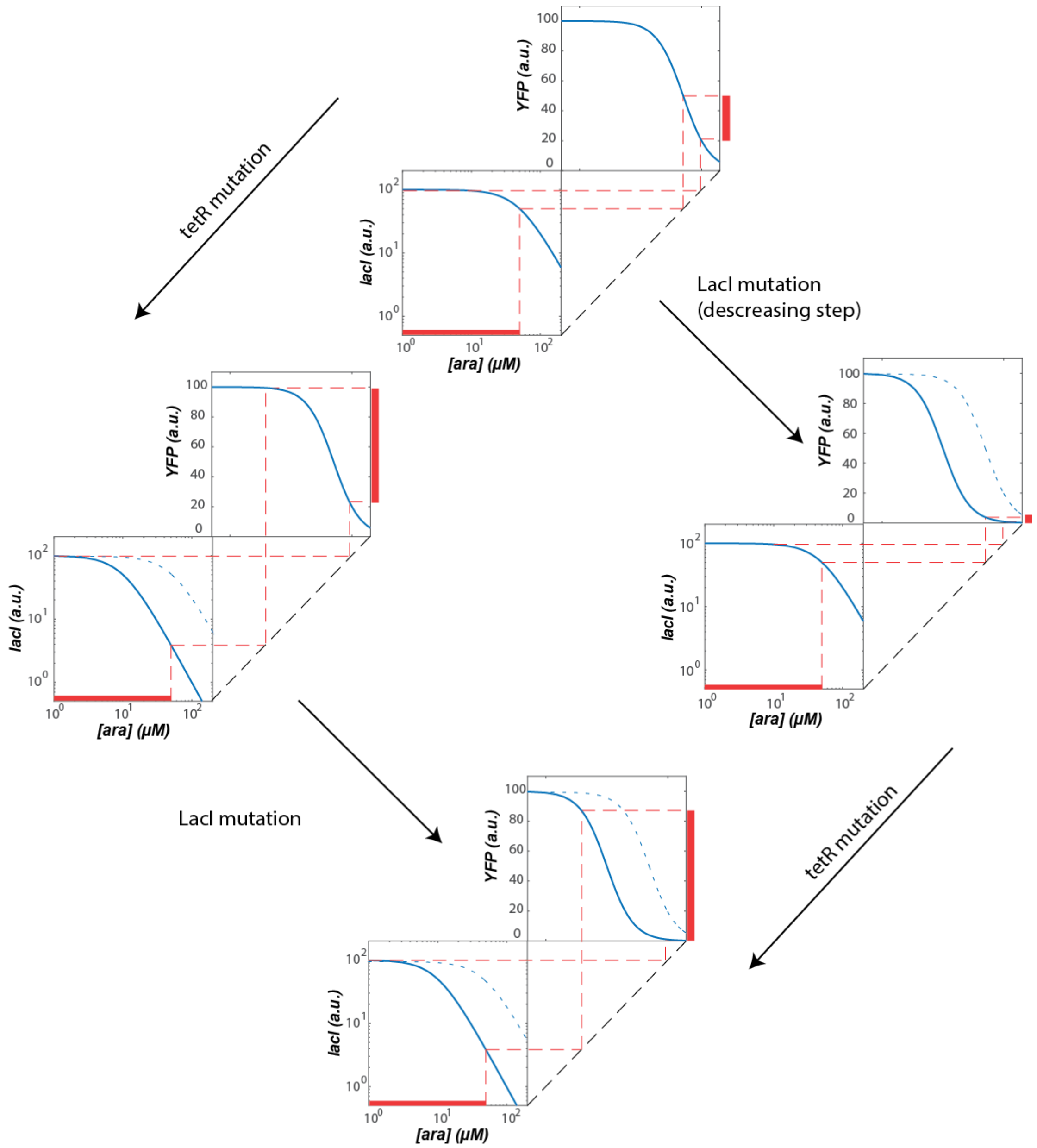

Supplementary Figure 6: Mechanistic decomposition of downstream sign epistasis. The four stair-like diagrams decompose the full input-output relation into the responses of *tetR* and *lacI*. Diagrams were obtained using the cascade numerical model with a cooperativity of 2 and a 5 fold change in dissociation constants due to mutations. The (small) arabinose input range (thick red horizontal bars) remains the same. Red dashed lines indicate how *tetR* and *lacI* transduce signals varying within this input range. The output of the first component is reflected on an oblique line to become the input range for the second component. *tetR* mutations modify the *ara*-to-*lacI* relation. *lacI* mutations modify the *lacI*-to-*YFP* relation. Reponses before mutations are represented as blue dotted curves. The *YFP* output range (thick red vertical bars) changes after each mutation. In this example of downstream sign epistasis, the *YFP* segment length decreases when *lacI* only is mutated, but steadily increases along the path where *tetR* is mutated first.

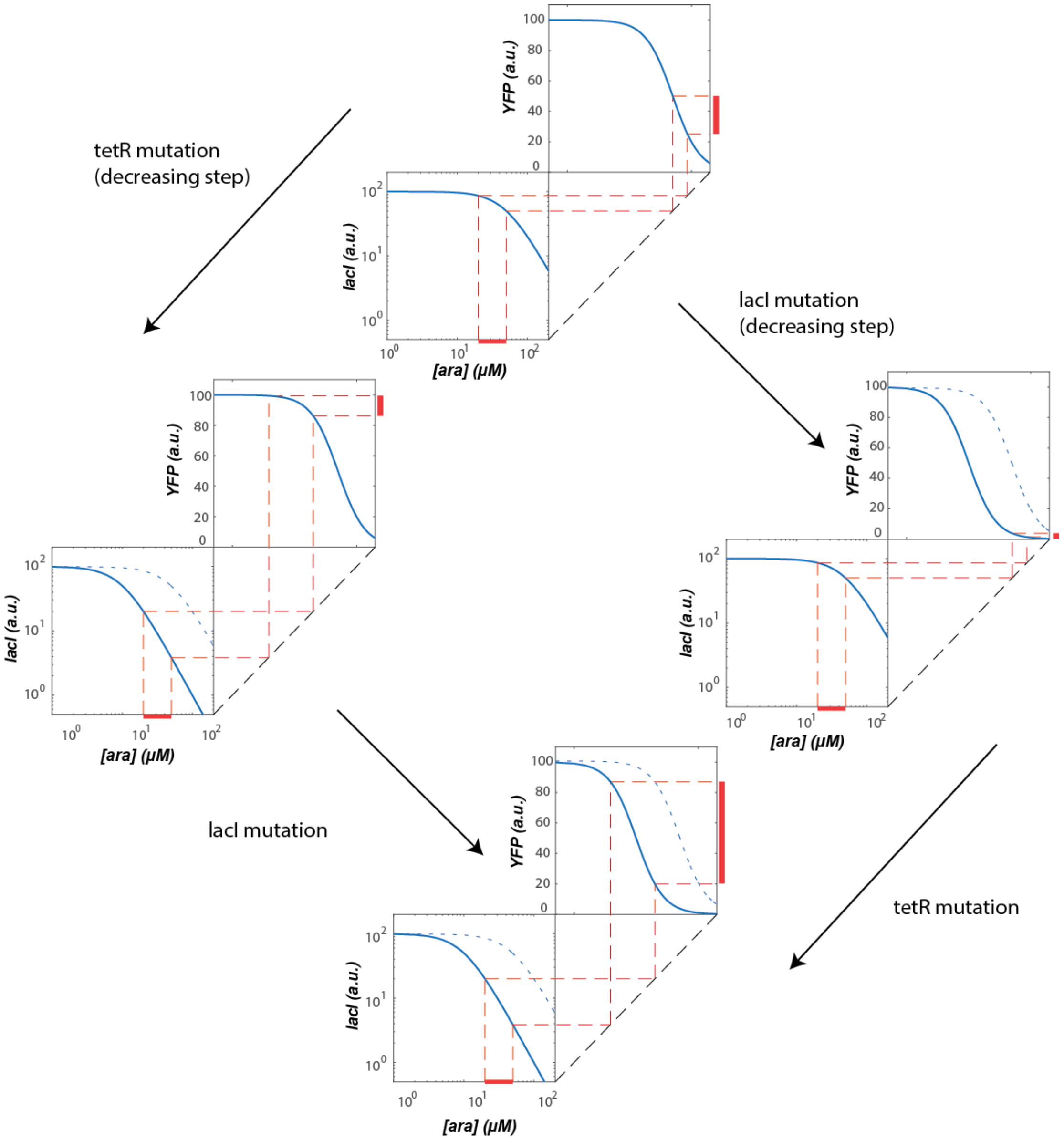

Supplementary Figure 7: Mechanistic decomposition of reciprocal sign epistasis. The four stair-like diagrams decompose the full input-output relation into the responses of *tetR* and *lacI*. Diagrams were obtained using the cascade numerical model with a cooperativity of 2 and a 5 fold change in dissociation constants due to mutations. The (small) arabinose input range (thick red horizontal bars) remains the same. Red dashed lines indicate how *tetR* and *lacI* transduce signals varying within this input range. The output of the first component is reflected on an oblique line to become the input range for the second component. *tetR* mutations modify the *ara*-to-*lacI* relation. *lacI* mutations modify the *lacI*-to-*YFP* relation. Reponses before mutations are represented as blue dotted curves. The *YFP* output range (thick red vertical bars) changes after each mutation. In this example of reciprocal sign epistasis, the *YFP* segment length decreases when *tetR* alone or *lacI* alone are mutated, but increases when both are mutated.

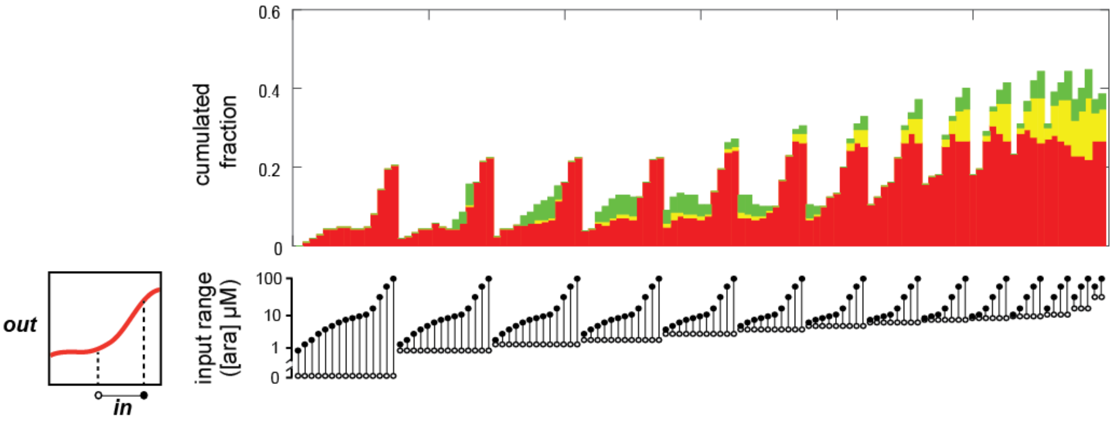

Supplementary Figure 8: Model prediction for epistasis fractions as a function of the input range. Top: Stack histogram of experimentally observed epistasis fractions, each stack corresponding to a different input range. Bottom: corresponding input ranges represented by vertical black lines (bottom: minimum input; top: maximum input). Refer to Fig. 2c for experimentally observed epistasis classes.

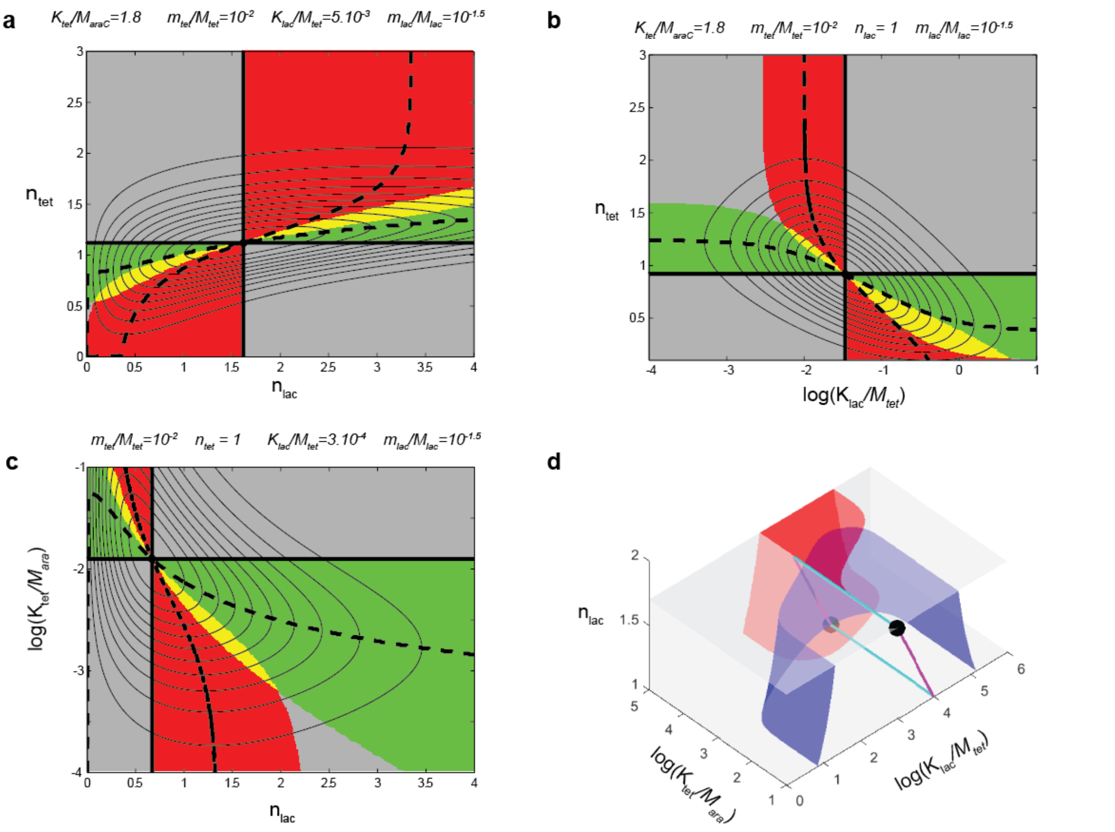

Supplementary Figure 9: Epistasis between other cascade parameters. Epistasis patterns obtained for mutations affecting: **a**) Hill coefficient of *tetR* versus Hill coefficient of *lacI*; **b**) Hill coefficient of *tetR* versus dissociation constant of *lacI*; **c**) Dissociation constant of *tetR* versus Hill coefficient of *lacI*. For each panel, the values of the fixed parameters are indicated on top, including the minimum expression level m_{tet} and m_{lac}, and the maximum expression levels M_{tetR} and M_{lacI}. **d**) To show how our approach holds for pleiotropic mutations that affect two parameters in one gene, we computed epistasis for the same parameter values as for Main Text Fig. 3 a, but also varying the *lacI* binding cooperativity. The red shades indicate the envelope of the downstream sign epistasis domain and the blue surface is a particular iso-fitness surface. Black dots are initial and final points of an example of double swap trajectory displaying sign epistasis, where purple lines correspond to *lacI* mutations (affecting both n_{lacI} and K_{lacI}) and cyan lines correspond to *tetR* mutations (affecting K_{tetR}). Starting in the bottom black dot, the first mutation upwards is seen to move away from the iso-fitness surface, and hence a decrease in fitness.

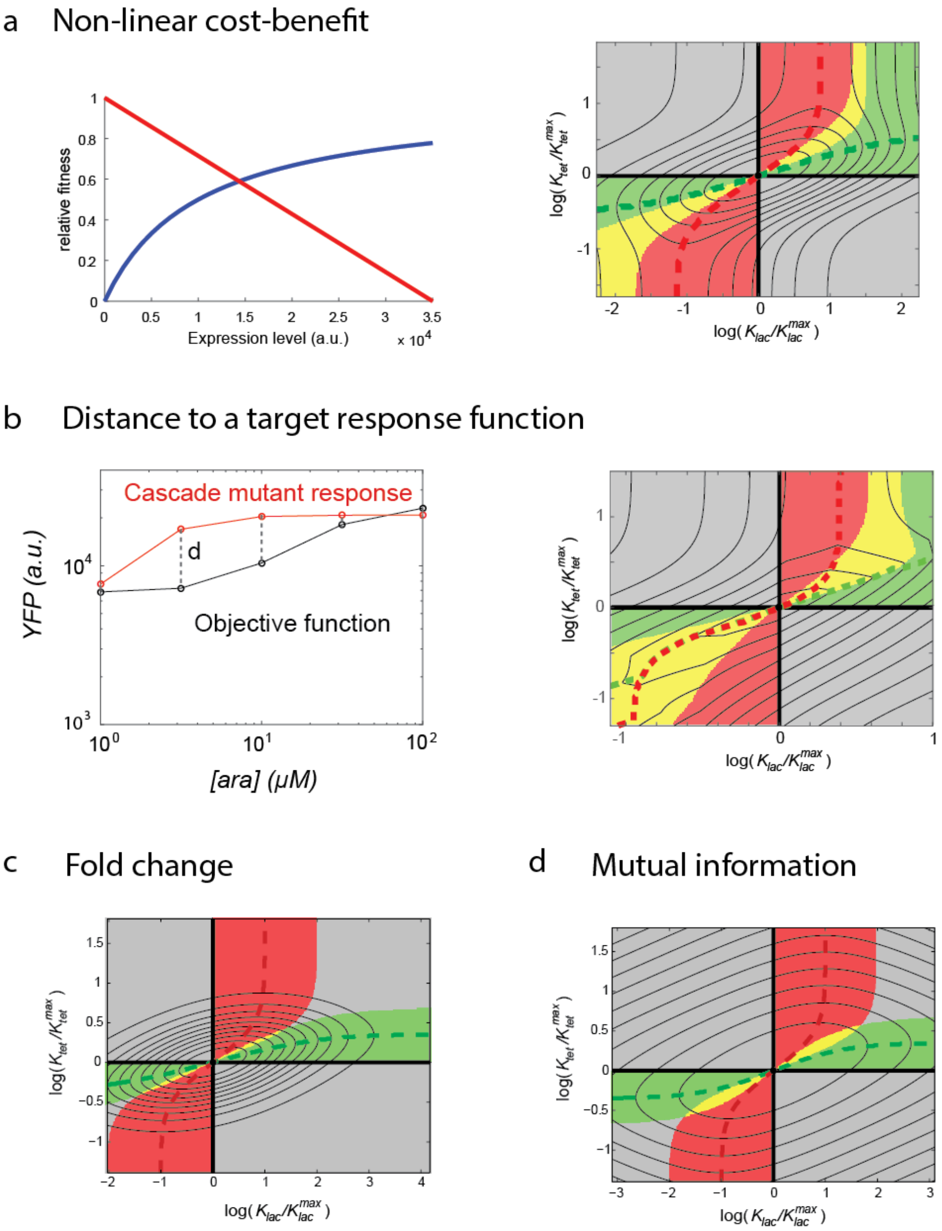

Supplementary Figure 10: Alternative fitness functions. All epistasis pattern were generated for the kinetic model using m_{tetR}=10^2, M_{tetR}=10^4, n_{tetR}=2.4, m_{lacI}=10^3, M_{lacI}=$10^{4.5}$, n_{lacI}=0.7, as a function of the dissociation constants K_{tetR} and K_{lacI} normalized to their optimum value. **a**) Linear cost function in environment 1 (red curve) and Monod-type saturating benefit function in environment 2 (blue curve). Sign epistasis patterns for trajectories ending at the fitness maximum when fitness is averaged between environments 1 and 2. **b**) Fitness penalty is the average of the Euclidian distance d between the cascade response (red curve) and an objective function (blue curve), computed at five logarithmically spaced values covering the experimental range. **c**) Fold-change of the output y_1/y_2. **d**) Performance measured as the mutual information between the input and the output $\mathrm{MI} = -\int_I P(c)\ln P(c)\,dc + \frac{1}{2}\int_I \ln\left(\frac{1}{2\pi e}\frac{1}{\sigma^2(c)}\left(\frac{dg}{dc}\right)^2\right)P(c)dc$ in the small noise approximation [4], with P the probability distribution of the input, σ^2 variance of the response, g the response function, P and σ^2 uniformly distributed.

Supplementary References

[1] Berge, C., Topological Spaces: including a treatment of multi-valued functions, vector spaces, and convexity, *Courier Corporation* (1963).
[2] Bintu, L., Buchler, N. E., Garcia, H. G., Gerland, U., Hwa, T., Kondev, J., & Phillips, R. (2005). Transcriptional regulation by the numbers: models. *Current opinion in genetics & development*, *15*(2), 116-124 (2005).
[3] Sontag, E. D., Monotone and near-monotone biochemical networks, *Systems and Synthetic Biology*, *1*(2), 59-87 (2007).
[4] Tkačik, G., Walczak, A. M., & Bialek, W., Optimizing information flow in small genetic networks. *Physical Review E*, *80*(3), 031920 (2009).
